# The infectious BAC genomic DNA expression library: a high capacity vector system for functional genomics

**DOI:** 10.1038/srep28644

**Published:** 2016-06-29

**Authors:** Michele M. P. Lufino, Pauline A. H. Edser, Michael A. Quail, Stephen Rice, David J. Adams, Richard Wade-Martins

**Affiliations:** 1 Department of Physiology, Anatomy and Genetics, University of Oxford, Le Gros Clark Building, South Parks Road, Oxford, OX1 3QX, UK; 2 Wellcome Trust Sanger Institute, Hinxton, Cambridgeshire, CB10 1SA, UK

## Abstract

Gene dosage plays a critical role in a range of cellular phenotypes, yet most cellular expression systems use heterologous cDNA-based vectors which express proteins well above physiological levels. In contrast, genomic DNA expression vectors generate physiologically-relevant levels of gene expression by carrying the whole genomic DNA locus of a gene including its regulatory elements. Here we describe the first genomic DNA expression library generated using the high-capacity herpes simplex virus-1 amplicon technology to deliver bacterial artificial chromosomes (BACs) into cells by viral transduction. The infectious BAC (iBAC) library contains 184,320 clones with an average insert size of 134.5 kb. We show in a Chinese hamster ovary (CHO) disease model cell line and mouse embryonic stem (ES) cells that this library can be used for genetic rescue studies in a range of contexts including the physiological restoration of *Ldlr* deficiency, and viral receptor expression. The iBAC library represents an important new genetic analysis tool openly available to the research community.

While DNA sequencing has identified a wealth of candidate disease genes, for many patients pathogenic variants cannot be confirmed from their DNA sequence alone. One approach for disease gene validation is genetic rescue using patient-derived cells *in vitro*. Cell-based rescue experiments are typically carried out using cDNA expression constructs^1^,^2^ with vectors containing strong heterologous promoters which assumes that genetic rescue of a phenotype is best achieved by overexpression, regardless of the potential for artefacts, or cellular toxicity, and irrespective of the requirement for *cis*-regulatory elements that may be necessary to express a gene in the right cellular context, or at a defined phase of the cell cycle. Furthermore, cDNA vectors may be silenced over time, lack the potential to express all splice variants of a gene and, since they are often constructed in lentiviral or retroviral backbones, they may integrate into the genome and cause insertional mutagenesis^3^,^4^. Genomic DNA expression vectors have advantages in that they may contain an entire gene and the regulatory elements that control its expression^5^,^6^,^7^. Obtaining gene expression from a genomic locus is a complex event involving the interaction of multiple promoter, enhancer and silencer elements to achieve gene expression correctly regulated in a developmental and tissue-specific manner. In many cases the elements regulating expression of a gene have not been defined, thus the delivery of the whole genomic locus represents the most suitable method of obtaining expression properties that resemble those of the gene in its endogenous locus. Previous studies comparing expression from cDNA and genomic DNA expression vectors delivered *in vivo* showed that transgene expression from cDNA vectors declines over time whereas genomic DNA vectors may retain sustained physiological expression levels for several months after delivery^8^,^9^. Furthermore, it has been shown that by delivering whole genomic loci feedback regulation of gene expression may be retained; an event critical for fine-tuning of expression in response to physiological cues. Genomic DNA vectors may also allow expression of alternative transcripts and differential promoter usage, key gene regulatory mechanisms for physiological control^10^,^11^.

Genomic DNA vectors are generally based on bacterial artificial chromosomes (BACs), which are highly stable, circular self-replicating vectors capable of carrying inserts of up to 300 kb of DNA sequence^12^,^13^. BAC vectors have been widely used in rodent transgenesis and have been shown to provide temporal and tissue-specific transgene gene expression^14^,^15^. Thus, BAC libraries are a valuable resource for use in functional studies, however they are currently underused *in vitro* due to the difficulties in delivering BACs efficiently to cells in culture. The delivery of whole BAC libraries to cell culture systems, such as human patient cell lines with a genetic disorder, would allow for high-throughput gene discovery via genetic rescue where a scorable phenotype from patient cells is observable. While viral vectors are an efficient means of delivering genes into cells in culture most viral systems do not have sufficient transgene capacity to carry a complete genomic DNA locus. An exception is the herpes simplex virus type-1 (HSV-1) amplicon system, which has a transgene capacity of up to ~150 kb. We have previously developed the infectious BAC (iBAC) system which is based on the HSV-1 amplicon technology to mediate the delivery of intact genomic DNA loci >100 kb both *in vitro* and *in vivo*^4^,^16^. Here we describe a genome-wide library of infectious DNA amplicons carrying DNA sequences derived from HSV-1, which allow vector packaging and delivery through high-capacity replication-deficient HSV-1 amplicon vectors^17^. The iBAC vector also carries Epstein-Barr virus-derived *EBNA-1/oriP* extra-chromosomal retention elements that ensure episomal maintenance of transferred loci^18^,^19^,^20^. The library of 184,320 clones has an estimated 90% coverage of the autosomes and has been characterized by end-sequencing and found to have an average genomic DNA insert size of 134.5 kb, which is the optimal size for HSV-1 amplicon packaging^16^. Clones of interest are publicly accessible and can be easily identified by the scientific community through the Ensembl browser. Here we show how clones from the iBAC library may be used for functional rescue in cell lines with genetic deficiencies.

## Results

### Construction and analysis of the iBAC genomic DNA expression library

**Figure 1:**
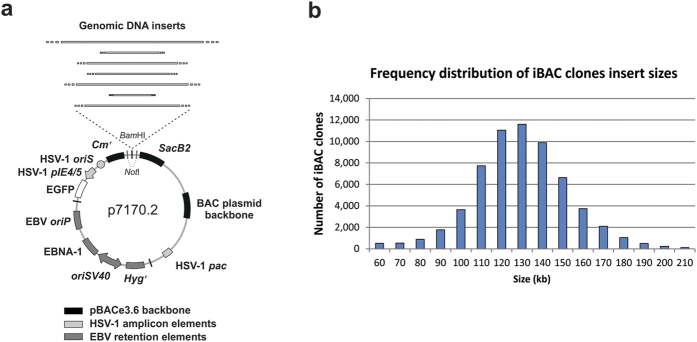
Construction of the iBAC library and characterisation of insert sizes of iBAC clones. (**a**) The iBAC library vector carries DNA elements for packaging into HSV-1 amplicon vectors, extrachromosomal retention and bacterial replication. Genomic DNA fragments were cloned into the unique Bam HI site. (**b**) Frequency distribution of iBAC clones insert sizes based on end-sequencing of 62,825 clones. The iBAC library clones were subjected to BAC end sequencing using the universal primers T7 and SP6 and the sequences were mapped onto the mouse genome. Each bar represents the number of iBAC clones falling within a + 10 kb interval (i.e. 100 kb = all clones between 100,000 bp and 109,999 bp).

To construct the iBAC library, we performed a partial Mbo I digest on male C3H/HeJ mouse genomic DNA and subcloned digested fragments into the iBAC library vector using a unique Bam HI cloning site (Fig. 1a). The iBAC vector contains sequences for bacterial replication, extra-chromosomal retention and packaging into HSV-1 amplicons. The entire library consists of 184,320 iBAC clones (C3H) arrayed in 480 × 384-well plates.

We paired end-sequenced 87,120 iBAC clones and uniquely mapped 62,825 iBAC inserts to the mouse genome (m38), identifying an average genomic DNA insert size of 134.5 kb (Fig. 1b), which is the optimal size for HSV-1 amplicon packaging^16^. Since iBAC clones with a vector size <80 kb would be packaged at two or more copies of vector per HSV-1 viral particle^21^,^22^,^23^, we quantified the total number of clones smaller than 80 kb and found that only 1557 out of 62,825 iBAC clones fall within this category, accounting for only 2.5% of the library.

The 62,825 clones identified and mapped by end-sequencing provide 90.2% average coverage of the autosomes at a depth of 3.3-fold (Table 1) and we estimate a 7–8 fold depth of genome coverage for the whole library of 184,320 clones. In order to allow public accessibility of the library, the clones may be displayed on the Ensembl genome^24^ by uploading the .bed file provided (Supplementary Fig. S1).

### iBAC library clone stability in *E. coli*

**Figure 2:**
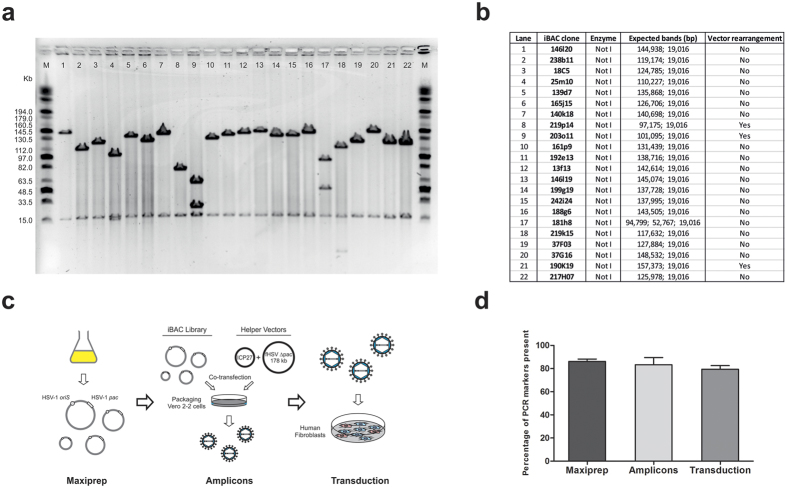
Analysis of stability of the iBAC library in *E. coli* and assessment of retention of coverage during the three stages of preparation of the iBAC library. (**a**) Analysis of vector stability was carried out by performing a Not I digestion of 22 iBAC clones with a genomic insert <150 kb. Lanes: M = Midrange I PFG marker; lanes 1–22 = iBAC library clones described in (**b**). (**b**) Expected fragment size of digestions of the iBAC library clones shown in (**a**) was obtained by mapping BAC end reads to the mouse genome using the Ensembl. Vector rearrangement was detected in three out of 22 clones, suggesting a frequency of rearrangement of 13.6%. (**c**) Schematic representation of the three stages involved in the preparation and delivery of the iBAC library, namely: vector DNA preparation, packaging into HSV-1 amplicons and viral transduction of target cells. (**d**) Genome coverage was assessed by STS marker PCR at the three stages described in (**c**) after preparation and delivery to cells of the whole library made of 184,320 clones. No significant difference was observed between the different stages of library preparation using one-way ANOVA test with Dunnett’s multiple comparisons test (compared to Maxiprep). Error bars represent mean +/−SEM.

We next analyzed the stability of iBAC library clones in *E. coli* by restriction enzyme digestion analysis, since a high frequency vector rearrangement would interfere with the functionality of the library as a molecular tool. We randomly selected 22 iBAC clones with inserts <150 kb and performed a Not I digest, which is expected to release the genomic DNA insert due to the presence of Not I sites flanking the unique Bam HI cloning site in the p7170.2 vector (Fig. 1a). The expected size of the fragments was predicted by mapping the BAC ends to the mouse genome using the Ensembl browser. Fragments of predicted size were identified in 19 out of the 22 clones, revealing an estimated frequency of BAC vector rearrangement, at this resolution, of 13.6% (Fig. 2a,b). This low frequency of gross rearrangement is consistent with that of previously reported BAC libraries^25^ and we believe it is unlikely to interfere with the use of the iBAC library for functional studies, considering its coverage of the autosomes at an approximate depth of 7–8-fold (Table 1).

### Retention of the iBAC library during the preparation and packaging into HSV-1 amplicons

To assess the retention of the iBAC library through the process of vector preparation and delivery of the library to cells in culture, we assayed library genome coverage at the stages of DNA maxiprep (DNA preparation prior to packaging), amplicon packaging (performed in Vero 2.2 cells), and vector transduction into human fibroblasts in culture (Fig. 2c). To carry out this analysis, we selected 94 PCR primer pairs evenly spread across the mouse genome (Supplementary Fig. S2a)^26^ and used a nested-PCR protocol to ensure sensitive detection of iBAC DNA at each stage of the procedure (Supplementary Fig. S2b,c). The primer pairs were selected to be mouse-specific by a sequence comparison against the human and mouse genomes. Furthermore, the primer pairs were selected to amplify regions known to be polymorphic between 129 (substrain 129S7/SvEvBrd) and C3H mice to facilitate the identification of C3H iBAC library clones in recipient mouse cell lines (eg: 129 embryonic stem (ES) cells). For this experiment we delivered the entire 184,320-clone iBAC library into human fibroblasts and observed equal levels of retention of the library throughout packaging and delivery (Fig. 2d).

### Physiological expression and phenotype correction of a cell line deficient for low-density lipoprotein receptor function using individual iBAC library clones

**Figure 3:**
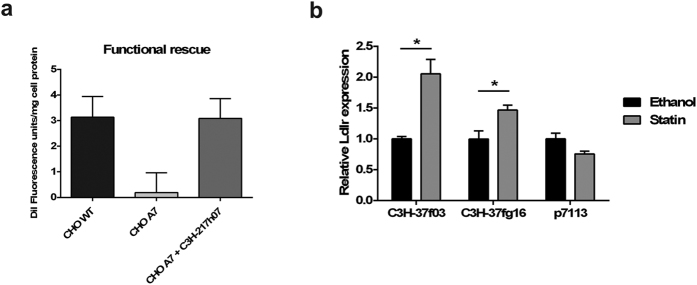
Functional complementation of *Ldlr* deficiency and physiological regulation of *Ldlr* transgene expression using individual iBAC library clones. (**a**) The iBAC *Ldlr* clone C3H-217h07 was packaged into HSV-1 amplicons and purified HSV-1 amplicons were used to transduce *Ldlr* deficient CHO *ldlr*^−/−^ a7 cells. Cells infected with C3H-217h07 show restoration of Ldlr function to wild-type levels. Error bars represent mean +/−SEM. (**b**) CHO *ldlr*^−/−^ a7 cells were transfected with either an iBAC clone (C3H-37f03 or C3H-37g16) or with the pCMV-LDLR plasmid p7113 and incubated with simvastatin at 300 nM for 48 hr. *Ldlr* expression was quantified by qRT-PCR and normalized by transfection efficiency. iBAC *Ldlr* clones show a significant 1.5–2.0 fold increase in expression following statin treatment whereas p7113 shows no up-regulation. Error bars represent mean ± SEM. *P < 0.05, as determined by unpaired two-tailed Student’s t-test.

To assess the functionality of the iBACs in the library we selected a clone covering the complete low-density lipoprotein receptor (*Ldlr*) locus (clone C3H-217h07) (Supplementary Fig. S1). The regulation of *Ldlr* transcription by *cis*-genomic DNA sequences is well characterised making this gene an excellent candidate for assessment of the iBAC library as an expression tool^11^,^27^. The iBAC library clone C3H-217h07 was packaged into HSV-1 amplicons using an improved helper-virus free packaging system (Fig. 2c)^17^, and used to transduce *Ldlr*-deficient CHO *ldlr*^−/−^ a7 cells at an MOI of 10. Ldlr function was assayed using fluorescently-labelled low density lipoproteins (DiI-LDL). The iBAC clone C3H-217h07 correctly restored Ldlr activity in Chinese hamster ovary (CHO) *ldlr*^−/−^ a7 cells to wild-type levels, confirming the functionality of genomic DNA sequences included in the iBAC vector (Fig. 3a).

To investigate whether iBAC library clones are capable of achieving physiologically-relevant regulation of expression, we further analysed two *Ldlr* clones in detail. We transfected CHO *ldlr*^−/−^ a7 cells with either an iBAC clone carrying the full *Ldlr* genomic DNA locus (C3H-37f03 or C3H-37g16) or p7113, a plasmid carrying the human *LDLR* cDNA gene under the control of the immediate early promoter of the cytomegalovirus (pCMV), thus lacking the necessary regulatory elements. Since *Ldlr* expression is finely regulated by intracellular sterol levels through a negative feedback mechanism^11^,^27^, we then incubated the transfected cells with simvastatin, as statins are known to induce *LDLR* up-regulation from the genomic locus by reducing intracellular cholesterol synthesis^28^. As shown in Fig. 3b *Ldlr* expression from both C3H-37f03 and C3H-37g16 iBAC clones is significantly increased after incubation with simvastatin, whereas the *pCMV-LDLR* vector, as expected, fails to up-regulate *LDLR* expression. These data demonstrate the advantages of using the genomic DNA iBAC library by showing successful physiological regulation of gene expression from genomic DNA library clones.

### Phenotype rescue and extrachromosomal iBAC vector retention in dividing cells

**Figure 4:**
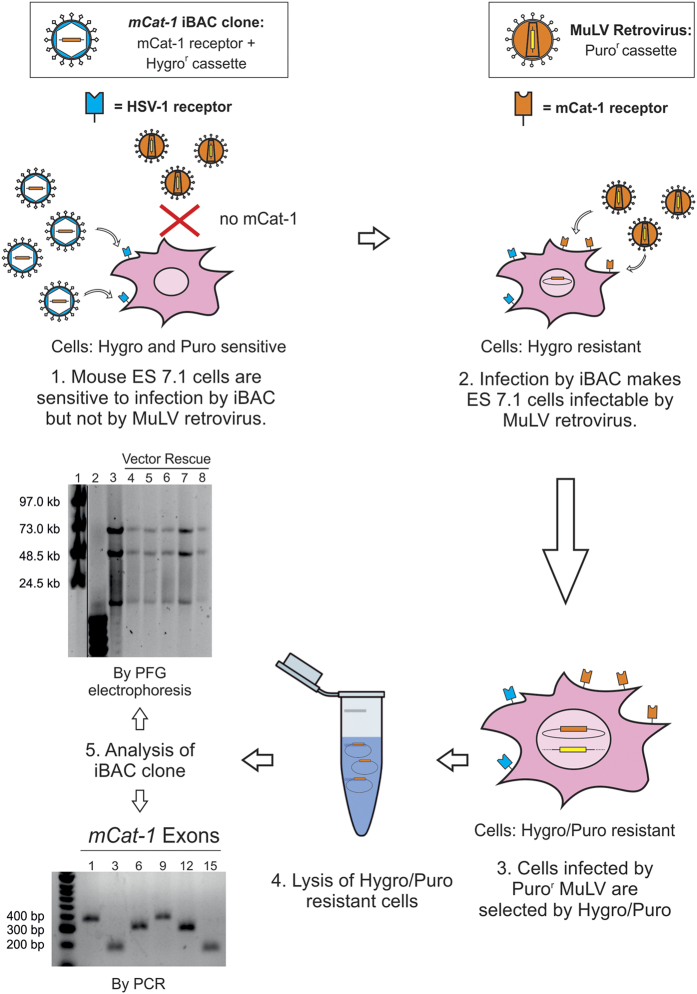
Phenotype rescue and extrachromosomal vector retention after culture in dividing cells. Mouse ES 7.1 cells are uninfectable by MuLV retroviruses since they lack expression of *mCat-1* receptor gene. However, transduction with the iBAC clone C3H-17l06 carrying the whole *mCat-1* genomic DNA locus allows successful MuLV retroviral transduction. After the double infection by C3H-17l06 HSV-1 amplicons and Puro^r^ MuLV retroviruses, cells were positively selected by hygromycin and puromycin and the episomal *mCat-1* iBAC DNA was rescued back into bacteria and analysed by PCR and by pulsed-field gel electrophoresis (PFG) after Not I digestion. PFG: lane 1 = Midrange II PFG marker; lane 2 = 1 kb Plus DNA Ladder; lane 3 = C3H-17l06 DNA; lanes 4–8 = Rescued iBACs. PCR: lane 1 = HyperLadder 50 bp; lanes 2–7 = *mCat-1* exons 1, 3, 6, 9, 12, 15.

To assess whether clones from the library can be maintained and successfully identified after a selection process which is an essential requirement for long term functional studies, we took advantage of an assay in which retroviral transduction of ES 7.1 cells by the ecotropic murine leukemia virus (MuLV) is dependent on expression of a functional *mCat-1* receptor gene, encoded by the *Slc7a1* gene^29^,^30^,^31^. The ES 7.1 cell line does not express the *mCat-1* receptor due to the integration of a retroviral gene-trap vector carrying a splice-acceptor site downstream of exon 2 of *Slc7a1* and, thus, is not susceptible to MuLV infection^29^. ES 7.1 cells were transduced by an individual iBAC covering the *mCat-1* gene (C3H-17l06), and forty-eight hours later the cells were super-infected with Puro/TK, a MuLV retrovirus carrying a puromycin resistance cassette (Fig. 4)^29^. Twenty-four hours later, puromycin/hygromycin double selection was applied to select for the presence of the retrovirus and the iBAC vector, respectively, and episomal DNA was extracted from cells under double selection after 14 days. Plasmid rescue demonstrated intact vector recovery of the C3H-17l06 iBAC clone into the bacterial host and PCR amplification of *Slc7a1* exons from the rescued BAC plasmid confirmed clone identity (Fig. 4). The experiment described here demonstrates successful functional rescue of *mCat-1* deficiency and extrachromosomal retention of the iBAC library clone in the absence of vector rearrangement after 14 days in dividing cells in culture.

## Discussion

Novel efficient approaches are urgently required to better understand the complex biological function of the genome and the role that mutations play in disease processes. Expression libraries represent an attractive platform for both forward and reverse functional analysis by allowing the identification of novel genes involved in a pathway of interest and a detailed analysis of gene function^32^. Among the different expression libraries currently available, those based on genomic DNA expression vectors represent an extremely promising tool for functional genomics studies due to their ability to provide physiological regulation of gene expression resembling that of a native endogenous locus^5^,^6^,^10^,^11^,^12^,^33^. This attractive feature derives from the high capacity of BAC vectors which allows the delivery of the complete genomic DNA locus of a gene of interest^13^. However, the use of genomic DNA libraries for functional screens has so far been hampered by the low efficiency of delivery of large vectors>100 kb to cells, since delivery of such libraries by non-viral methods is highly inefficient. Viral vectors provide an attractive alternative to the use of non-viral approaches, however most of the viral delivery systems available are characterized by limited transgene capacity.

Here we describe a major technical and conceptual advance by generating the first genomic DNA library which can be delivered by viral transduction. The iBAC library contains sequences that allow vector packaging into HSV-1 amplicons, a viral vector delivery system derived from the widespread human herpes simplex virus type-1^22^. HSV-1 amplicons are bacterial vectors carrying the HSV-1 lytic origin of replication (*oriS*) and the DNA cleavage/packaging terminal repeats, and lacking the vast majority of the HSV-1 genome, therefore allowing a maximum transgene capacity up to ~150 kb. Since these ‘gutless’ vectors carry only a few kilobases of HSV-1 DNA, packaging into HSV-1 amplicons requires co-transfection of both the vector to be packaged and a BAC containing the whole HSV-1 genome but lacking packaging signals, which provides helper function without contamination by recombinant HSV-1 viruses^34^. HSV-1 amplicons are able to efficiently infect a wide variety of dividing and non-dividing cells, therefore allowing delivery to cells of BAC vectors >100 kb in size at high efficiency. The iBAC library is composed of 184,320 clones with an average insert size of 134.5 kb, which is the ideal size for HSV-1 amplicons. We assessed the frequency of vector rearrangement of the iBAC library in *E. coli* and we confirmed a high level of stability, in line with previously reported BAC libraries^25^. We show that the library has at least 90% coverage of the autosomes at an estimated depth of 7–8-fold and we demonstrate retention of such coverage through the stages required for the preparation and delivery of the library. Furthermore, we show the functionality of our library by selecting iBAC clones through the Ensembl browser and utilizing them in two different *in vitro* functional rescue assays, in both cases demonstrating that individual iBAC clones are able to re-establish the physiologically-regulated expression observed at endogenous loci.

An important feature of the iBAC library is represented by the presence of the EBV episomal retention elements which allow vector replication and extrachromosomal maintenance in transduced cells. EBV-based BAC vectors have been shown to be retained in the absence of selection with an efficiency of 92–98% per cell division^7^,^18^,^35^, allowing vector retention during long-term cell culture followed by vector isolation and identification. Plasmid vectors based on the *EBNA-1/oriP* system and lacking genomic DNA sequence do not replicate in rodent cells^20^,^36^, however the inclusion of fragments of mammalian genomic DNA > 10 kb provides vector replication in a once-per-cell cycle fashion, due to the presence of mammalian replication origins^37^,^38^. By carrying genomic DNA fragments, we expect the iBAC library to be capable of vector replication, as demonstrated by the successful vector rescue after a prolonged selection screen in replicating cell lines (Fig. 4). This feature makes the iBAC library suitable for long term genetic screens.

In summary, the iBAC genomic DNA expression library represents a novel tool for functional studies by ensuring physiological transgene expression and efficient delivery to cells through the HSV-1 amplicon system. We believe the iBAC library is highly complementary to the new CRISPR technology which, although allowing the precise genetic modification of endogenous loci, is highly laborious and time consuming. Genomic DNA expression libraries such as the iBAC library are well-suited for the analysis of multiple wild-type or recombinant genomic DNA transgenes in a more time-effective manner. The iBAC library is a freely available resource and clones of interest are publicly accessible and can be easily identified by the scientific community through use of the .bed file provided in this study, making the iBAC library highly suitable for reverse functional studies. Finally, due to the long-term retention of the iBAC library in an extrachromosomal state and without vector rearrangement, we believe the library could represent a novel molecular tool for forward genetic screens.

## Methods

### iBAC library construction

The iBAC library vector p7170.2 is based on pBACe3.6 with the addition of the following sequences: the HSV-1 *ori*_*s*_ and *pac* sequences for packaging into HSV-1 virions; the EGFP reporter gene under the control of the strong HSV-1 IE4/5 promoter to enable tracking of vector delivery, and vector titration; and elements of the EBNA-1/*oriP* episomal retention system from the Epstein-Barr virus together with the hygromycin resistance gene for *in vitro* selection of cells. For library construction genomic DNA from a male C3H/HeJ mouse was partially digested with Mbo I to generate cohesive ends. Genomic DNA inserts were selected by size fractionation using pulsed-field gel electrophoresis to cluster around 140 kb, the optimal size for HSV-1 amplicon packaging, and ligated into Bam HI linearized p7170.2 vector. Following transformation into ElectroMax DH10B cells 184,320 clones were picked and arrayed into 480 × 384 well plates. The iBAC expression library is an openly available resource and further information on obtaining iBACs is available from https://www.sanger.ac.uk/form/-jfZxIjTNSYSlIfmEq0elKA.

### Analysis of iBAC clone insert sizes

The iBAC library clones were end-sequenced using the T7 and SP6 primer sequences flanking the inserts in p7170.2. BAC end-sequencing was performed as described previously^39^. The end-sequences were mapped to the mouse genome (GRCm38 assembly)^40^. Mapped clones were assembled from mapped end-pairs by checking several criteria, such as verifying that the separation distance between clone ends was consistent with the known range of BAC insert sizes.

### Assessment of the genome coverage of the transduced iBAC library by PCR

To assess genome coverage of the iBAC library during library preparation, primer pairs from a previously described Sequenom platform assay selected to be polymorphic between the mouse strains 129 (substrain 129S7/SvEvBrd) and C3H/HeJ^26^ allowed us to detect iBACs in library preparations and in transduced cells. Each primer was confirmed for its specificity to the mouse genome by using the BLAST algorithm against both the mouse and human genome databases and primer pairs were designed to produce a PCR product between 200 and 300 bp in length. The sequence of the M13 forward (5′-GTAAAACGACGGCCAGT-3′) or reverse (5′-CAGGAAACAGCTATGAC-3′) universal primers sequence was added to the 5′ end of forward or reverse primers, respectively. Primer sequences are available on request. Each primer pair was optimised using AmpliTaq Gold (Life Technologies) by adjusting magnesium chloride concentration and the annealing temperature. Two rounds of PCR were carried to analyse the genomic coverage of the iBAC library using sequence tagged sites (STSs). In the first round the polymorphism-specific primer pairs carrying the M13 universal primer sequences were used to identify the STS and add the M13 tags onto the ends of the PCR product. In the second round the forward and reverse M13 primers were used to amplify the product further. DNA preparations of the iBAC library were performed from 250 ml *E. coli* cultures using a modified Tip 500 Maxiprep Kit protocol (Qiagen)^41^. DNA was extracted from amplicon preparations using the GenEluteTM (Sigma) mammalian genomic DNA kit. Total genomic DNA was extracted from transduced MRC-5V2 fibroblasts by addition of lysis buffer (0.65% SDS, 100 mM NaCl, 50 mM Tris.Cl pH 8, 20 mM EDTA, 100 μg/ml proteinase K) to cells in a 10-cm dish, incubation of cell lysate for 16 hr at 37 °C, followed by phenol/chloroform extraction, DNA precipitation and resuspension in TE (10 mM Tris, 1 mM EDTA).

### HSV-1 amplicon vector packaging

HSV-1 amplicons were produced using an improved helper virus-free packaging system as previously described^17^. Typically, the supernatant from three 6-cm dishes was concentrated by ultracentrifugation at 22,000 r.p.m. for 3 hr in a SW41 rotor (Beckman), and resuspended in 250 μl of DMEM, 10% FBS, P/S, L-glutamine, to give an average stock of 1–2 × 10^7^ transducing units (t.u.)/ml. For amplicon titration 4 × 10^5^ G16.9 cells were seeded per well of a 24-well plate and infected 24 hr later with iBAC amplicons. Twenty-four hours after infection, titre was determined by GFP reporter gene expression analysis.

### Low density lipoprotein receptor *(Ldlr)* expression analysis

Ldlr function was assayed by DiI-LDL uptake^6^. 1 × 10^4^ CHO wild-type and CHO *ldlr*^−/−^ a7 cells were seeded per well of a 24-well plate and 24 hr later the medium was replaced with Ham’s F12 medium supplemented with 5% lipoprotein-deficient FBS (LPDS, Biomedical Technologies, Inc, Stoughton, MA, USA). After forty-eight hours, cells were transduced with HSV-1 amplicons at a multiplicity of infection (MOI) of 10 and 24 hours later the infection mix was replaced with fresh LPDS-containing media. After 72 hr, media containing DiI-LDL (AbD Serotec, Kidlington, Oxford) at a concentration of 10 μg/ml was added to cells and cells were incubated for 5 hr at 37 °C. The DiI-LDL mix was then removed, cells were washed once in Ham’s F12 medium and DiI fluorescence was qualitatively analyzed on an Eclipse TE2000-U (Nikon) inverted microscope. For quantitative analysis, cells were then washed twice with PBS with Ca^2+^ and Mg^2+^ containing 0.4% bovine serum albumin (BSA), and three times with PBS alone and lysed with 0.1% SDS/0.1N NaOH. Fluorescence in the cell lysate was analyzed with a Jenway 6280 fluorimeter at excitation and emission wavelengths of 520 and 580 nm, respectively, and total protein content determined using Bicinchoninic acid assay (BCA, Sigma). Non-specific binding was determined in the presence of a 50-fold excess of unlabelled LDL (AbD Serotec, Kidlington, Oxford) and subtracted from total binding to give specific binding.

*Ldlr* expression following incubation with simvastatin was assessed by quantitative real-time PCR (qRT-PCR). 3.5 × 10^5^ CHO *ldlr*^−/−^ a7 cells were seeded per well of a 6-well plate and on the next day they were transfected with either 452 ng of iBAC (C3H-37F03 or C3H-37G16) per well or 113 ng of p7113 (a 14.2 kb vector carrying a *CMV-LDLR* cassette and elements for HSV-1 amplicon packaging) per well. The DNA quantities used were identified as able to achieve comparable transfection efficiency. Twenty-four hours after transfection, the medium was replaced with Ham’s F12 medium supplemented with 5% LPDS. After 24 hours, the medium was replaced with Ham’s F12 medium with 5% LPDS supplemented with simvastatin (Sigma, S6196) at a final concentration of 300 nM. Since simvastatin was resuspended in absolute ethanol an equivalent amount of ethanol was added to the medium in control incubations. After 24 hours, cells were washed in PBS and total RNA was extracted using RNeasy Mini Kit (Qiagen) and treated with RNase-Free DNase (Qiagen). cDNA was synthesized from 1 μg of total RNA using random primers (Life Technologies) and SuperScript III Reverse Transcriptase (Life Technologies) in a reaction volume of 20 μl. qPCR was carried out as described in^42^, using: RNA-mLdlr 2F (5′-GAGGAACTGGCGGCTGAA-3′) and RNA-mLdlr 2R (5′-GTGCTGGATGGGGAGGTCT-3′) for *mLdlr* mRNA detection; p7113 1F (5′-GGATGACGTGGCGTGAAA-3′) and p7113 1R (5′-TTAAACGGGCCCTCTAGACT-3′) for detection of *CMV-LDLR* mRNA expression; and finally, EGFP 2F (5′-TATATCATGGCCGACAAGCA-3′) and EGFP 2R (5′-GAACTCCAGCAGGACCATGT-3′) for EGFP mRNA detection. *mLdlr* and p7113 data were normalized to EGFP to account for variability in transfection efficiency between iBAC and p7113 vectors.

### Retroviral vector production

Production of Puro/TK MuLV retroviral vectors was performed using the stable helper-free retroviral producing cell line Phoenix Eco, as previously described^29^,^43^.

### Rescue of ES 7.1 retroviral transduction by delivery of the *mCat-1* locus

ES 7.1 cells are not infectable by MuLV retroviruses since they lack expression of the *Slc7a1* gene^29^, which encodes a cationic amino acid transporter which acts as a murine ecotropic leukaemia virus receptor^30^,^31^. The generation of these cells by genome-wide insertional mutagenesis in Blm-deficient ES cells has been previously reported^29^. 1 × 10^4^ ES 7.1 cells, which are *mCat-1* null, were seeded per well of a 24-well plate and 24 hr later cells were transduced with the iBAC C3H-17l06 HSV-1 amplicons. After 24 hours, the infection mix was removed and replaced with fresh media. 24 hours later, an infection mixture consisting of Puro/TK MuLV retroviral preparation and polybrene (hexadimethrine bromide; Sigma) at a final concentration of 10 μg/ml was added to the cells. Finally, after a further 24 hours the media was changed and hygromycin B (Life Technologies) and puromycin (Life Technologies) selection was applied, until isolated colonies appeared.

### Episomal rescue of Hygromycin/Puromycin-resistant ES 7.1 cells

Episomal DNA was extracted using an alkaline lysis method as described in ref. 6 and resuspended in 20 μl of TE with RNAse A 50 μg/ml. 10 μl of the episomal preparation were used to transform by electroporation DH10B bacteria, which were plated on LB agar with antibiotics. Plasmid DNA was prepared from the resulting bacterial colonies, digested with Not I and the digests were resolved by pulsed-field gel electrophoresis using the following conditions: V/cm = 6, run time = 16 hr, initial switch = 2 sec, final switch = 16 sec.

## Additional Information

**How to cite this article**: Lufino, M. M. P. *et al*. The infectious BAC genomic DNA expression library: a high capacity vector system for functional genomics. *Sci. Rep.* **6**, 28644; doi: 10.1038/srep28644 (2016).

## Supplementary Material

Supplementary Information

## Figures and Tables

**Table 1:** Summary of iBAC library end-sequencing data.

Chromosome	Total chromosome length	Non-redundant clone length	Clone length	Passed-aligned sequence	Depth	Percentage of total coverage
1	195,471,971	177,869,173	639,612,357	9,033,820	3.2721	90.99472016
2	182,113,224	166,295,001	589,252,333	8,259,235	3.2356	91.31407228
3	160,039,680	144,820,597	514,081,134	7,244,251	3.2122	90.4904315
4	156,508,116	140,452,375	521,445,598	7,260,730	3.3317	89.74127259
5	151,834,684	137,468,669	531,754,536	7,409,728	3.5022	90.53838384
6	149,736,546	134,958,678	489,830,541	6,900,789	3.2713	90.13075405
7	145,441,459	128,522,946	459,719,073	6,378,699	3.1609	88.36747574
8	129,401,213	115,613,659	425,815,567	5,946,793	3.2907	89.34511224
9	124,595,110	113,053,198	418,783,089	5,928,377	3.3612	90.7364647
10	130,694,993	118,696,440	423,388,647	5,955,991	3.2395	90.81942412
11	122,082,543	112,363,495	404,516,604	5,621,871	3.3135	92.03895351
12	120,129,022	107,787,553	375,265,890	5,218,482	3.1239	89.72648841
13	120,421,639	109,047,652	396,122,289	5,537,297	3.2895	90.55486448
14	124,902,244	110,574,528	392,527,177	5,499,962	3.1427	88.52885621
15	104,043,685	94,868,410	353,397,080	4,975,248	3.3966	91.18132446
16	98,207,768	90,123,456	313,944,892	4,445,290	3.1967	91.76815423
17	94,987,271	85,107,919	309,970,413	4,325,582	3.2633	89.59928852
18	90,702,639	81,666,643	288,909,371	4,048,624	3.1852	90.03778049
19	61,431,566	53,791,313	209,041,291	2,943,355	3.4028	87.56298513
X	171,031,299	121,211,671	280,610,536	4,002,156	1.6407	70.87104624
Y	91,744,698	52,994,066	108,824,786	1,595,221	1.1862	57.76253795

62,825 end-sequenced iBAC library clones were mapped to the mouse genome, revealing a 90.2% average coverage of the autosomes at a depth of 3.3-fold, providing an estimate for the coverage of the whole library of 7–8 fold.
