# Supplementary material for: The infectious BAC genomic DNA expression library: a high capacity vector system for functional genomics
Source: Sci Rep. 2016 Jun 29;6:28644. doi: 10.1038/srep28644 (PMC4926088; doi:10.1038/srep28644)
Supplement: Supplementary Information [file srep28644-s1.pdf]

The infectious BAC genomic DNA expression library: a high capacity vector system for functional genomics

Michele M.P. Lufino, Pauline A.H. Edser, Michael A. Quail, Stephen Rice, David J. Adams and Richard Wade-Martins

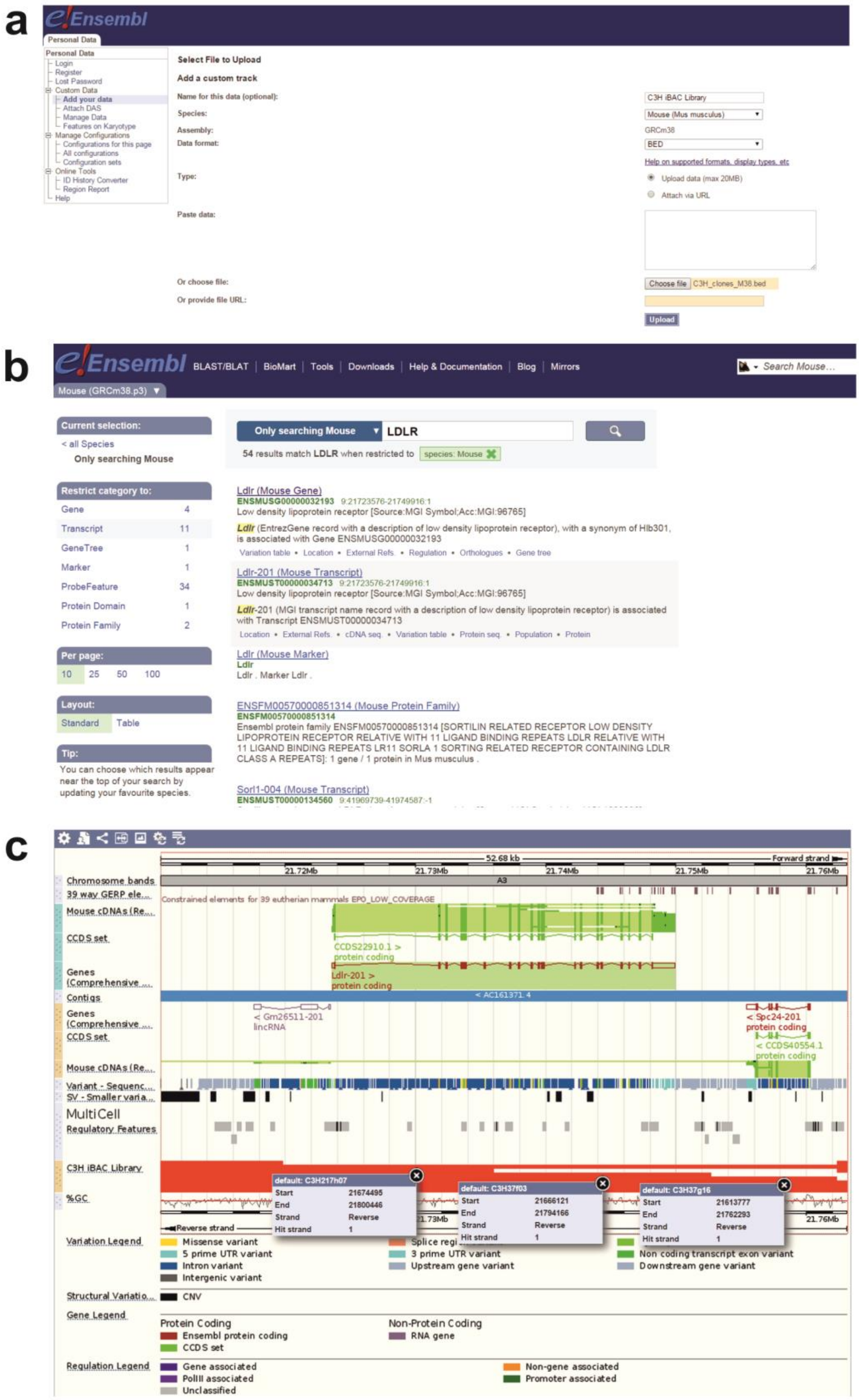

Supplementary Figure S1: iBAC library clones covering a gene of interest are identified using the Ensembl genome browser, through three simple steps. (**a**) The iBAC library file C3H_clones_M38.bed is uploaded through the 'Display your data in Ensembl' section of the Ensembl

browser. (**b**) The gene of interest is searched through the browser. (**c**) Individual iBAC clones are visualised as red lines and their identity shown by clicking on each line. Here, we identified the iBAC clones C3H-217h07, C3H-37f03 and C3H-37g16 covering the whole *Ldlr* genomic DNA locus.

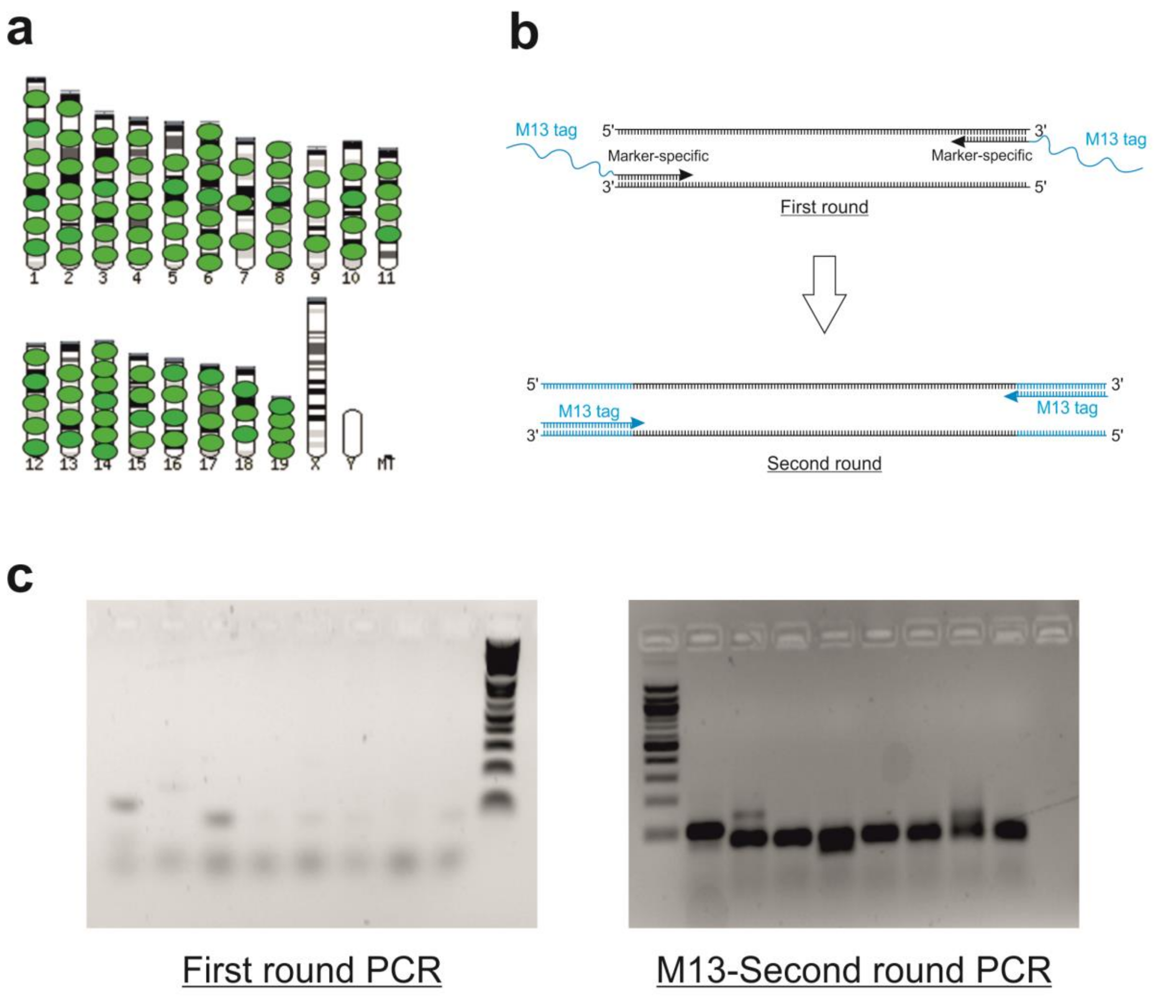

Supplementary Figure S2. Assessment of genome coverage by PCR. (**a**) Ninety-four previously published primer pairs selected to be polymorphic between human and mouse DNA and evenly spread across the genome were used[1]. (**b**) High sensitivity is achieved using a two-round PCR, by adding universal M13 sequences to the 5' end of the primers. (**c**) Robust detection of the entire iBAC library is achieved after two rounds of PCR.

REFERENCES

1 Moran, J. L. *et al.* Utilization of a whole genome SNP panel for efficient genetic mapping in the mouse. *Genome Res* **16**, 436-440, (2006).
